# Development of a novel WOrk-Related Questionnaire for UPper extremity disorders (WORQ-UP)

**DOI:** 10.1007/s00420-017-1246-7

**Published:** 2017-07-11

**Authors:** Bas R. J. Aerts, P. Paul F. M. Kuijer, Annechien Beumer, Denise Eygendaal, Monique H. W. Frings-Dresen

**Affiliations:** 1 Academic Medical Center, Department Coronel Institute of Occupational Health, Amsterdam Public Health research institute, University of Amsterdam, Meibergdreef 9, 1105 AZ Amsterdam, The Netherlands; 2 Department of Orthopaedic Surgery, Upper Limb Unit, Amphia Hospital, Breda, The Netherlands; 3 Department of Orthopaedic Surgery, Academic Medical Center, University of Amsterdam, Amsterdam, The Netherlands

**Keywords:** Upper extremity musculoskeletal disorders, Occupational diseases, Patient-reported outcome measurement, Limitations for work, Work participation, Upper limb

## Abstract

**Purpose:**

The aim of this study is to develop a patient-reported outcome measure (PROM) that identifies work-related limitations among patients with upper extremity musculoskeletal disorders in order to enhance work-directed care in daily orthopaedic practice, and to assess its content validity.

**Methods:**

The questionnaire was developed following the evaluation of existing PROMs and consensus within the research team. The content validity was assessed in three steps: (1) one on one interviews with patients (*n* = 14) were held to discuss the clarity and possible adaptation of items; (2) experts from the field (physiotherapists, insurance physicians, occupational health physicians, rehabilitation physicians and orthopaedic surgeons) were approached to participate in an interview to discuss the clarity, relevance and missing items; (3) patients (*n* = 12) were interviewed one on one to discuss the final version.

**Results:**

The first version of the WOrk-Related Questionnaire for UPper extremity disorders (WORQ-UP) consisted of 18 items based on the criteria: exertion, dexterity, handling tools & equipment, and mobility. According to patients (*n* = 14), 44% of the items were not easy enough to understand. Twenty-one experts [10 men, mean age 46 (SD = 8.5) and mean years of experience 16 (SD = 9.9)] participated in the interviews and adaptations were made. The final version of the WORQ-UP consisted of 17 items, all easy enough to understand according to patients (*n* = 12).

**Conclusions:**

A PROM specific for work-related limitations in patients with upper extremity musculoskeletal disorders was developed. According to patients and experts, it has sufficient content validity. The WORQ-UP can be used to assist in enhancing communication among healthcare workers to improve work-directed care and to evaluate effects of treatment on limitations at work.

**Electronic supplementary material:**

The online version of this article (doi:10.1007/s00420-017-1246-7) contains supplementary material, which is available to authorized users.

## Background

The worldwide point prevalence of upper extremity musculoskeletal disorders ranges from 2 to 53% and the 12-month prevalence ranges from 2 to 41% (Huisstede et al. 2006). In the Netherlands, 48% of the population aged 25 years and above reported upper extremity disorders during the past 12 months (Huisstede et al. 2008). Fifty-two percent of the general population in England reported symptoms of pain (lasting one day or longer) or dysesthesia (lasting at least 3 min) in the upper extremity or neck in the seven foregoing days. This corresponded to a 1 week prevalence of 24% for neck pain, 36% for upper limb pain, and 27% for sensory symptoms (Walker-Bone et al. 2004). In Europe, 25% of the workers reported work-related neck/shoulder pain, and 15% reported work-related pain of the arm (De Kraker and Blatter 2005).

To ensure that patients can return to work at an early stage and to enhance work participation, more attention is needed for the factor work in diagnosis and treatment of upper extremity disorders. In a number of clinics in the Netherlands, medical treatment has expanded to include return to work and continuing work. This work-directed care, however, is costly and is not covered by health insurance. Currently, no studies were found describing the effect of treatment programmes on return to work in patients with upper extremity musculoskeletal disorders visiting an orthopaedic clinic. As yet clinical practice is such that there is hardly any time or possibility during consultation for clinicians to enquire about limitations that patients (might) experience performing their work. Moreover, to support a patient-reported outcome measure (PROM) that quickly identifies work-related limitations might be of use. Eventually, this PROM could also aid in directing treatment approaches and potential return-to-work timeframes.

As yet, no questionnaire exists that reliably and validly determines the amount of limitations patients experience in work-related activities as a result of their upper extremity musculoskeletal disorders. The existing PROMs only have a few (1–5) general questions that ask if patients are experiencing limitations in performing their job (Table 1; Chung et al. 1998; Dawson et al. 1996; Hollinshead et al. 2000; Hudak et al. 1996; Kirkley et al. 1998, 2003; Lo et al. 2001; MacDermid et al. 1998; Michener et al. 2002).

**Table 1:** Overview of available PROMs regarding work and the upper extremity

PROM	Number	Content of the items
Western Ontario Osteoarthritis Shoulder index (WOOS)	5/19	How much difficulty do you experience working or reaching above shoulder level?
		How much difficulty do you experience with lifting objects (e.g. grocery bags, garbage can etc.) below shoulder level?
		How much difficulty do you experience doing repetitive motions below shoulder level such as raking, sweeping or washing floors because of your shoulder?
		How much difficulty do you experience pushing or pulling forcefully because of your shoulder?
		How troubled are you by an increase in pain in your shoulder after activities?
Rotator Cuff Quality of Life (RCQOL)	4/34	With respect to working with your arm at shoulder level, how much pain/difficulty do you experience because of your shoulder?
		With respect to working with your arm above shoulder level, how much pain/difficulty do you experience because of your shoulder?
		How much of the time are you concerned with missing days from work because of problems with or re-injury to your shoulder?
		How much of the time are you concerned that the activities you do at work may result in the state of your shoulder becoming worse?
Western Ontario Rotator Cuff index (WORC)	4/21	How much difficulty do you experience in daily activities about the house or yard?
		How much difficulty do you experience working above your head?
		How much do you use your uninvolved arm to compensate for your injured one?
		How much difficulty do you experience lifting heavy objects from the ground or below shoulder level?
Western Ontario Shoulder Instability index (WOSI)	1/21	How much has your shoulder affected your ability to perform the specific skills required for your sport or work? (If your shoulder affects both sports and work, consider the area that is most affected).
Oxford Shoulder Score	1/12	How much has pain from your shoulder interfered with your usual work (including housework)?
ASES Shoulder Score	1/17	Is it difficult for you to do your usual work?
Disabilities of the Arm Shoulder and Hand (DASH)	5/38	During the past week, were you limited in your work or other regular daily activities as a result of your arm, shoulder or hand problem?
		Doing your work as well as you would like?
		Using your usual technique for your work?
		Doing your usual work because of arm, shoulder or hand pain?
		Spending your usual amount of time doing your work?
Quick DASH	1/11	During the past week, were you limited in your work or other regular daily activities as a result of your arm, shoulder or hand problem?
Patient-Rated Wrist Evaluation (PRWE)	1/15	Rate the amount of difficulty you experienced performing your work (your job or usual everyday work)
Michigan Hand Outcome Questionnaire	5/37	How often were you unable to do your work because of problems with your hand(s) and/or wrist(s)?
		How often did you have to shorten your work day because of problems with your hand(s)?
		How often did you have to take it easy at your work because of problems with your hand(s) or wrist(s)?
		How often did you accomplish less in your work because of problems with your hand(s) or wrist(s)?
		How often did you take longer to do the tasks in your work because of problems with your hand(s) or wrist(s)?

*PROM* patient-reported outcome measure, *Number* amount of items per PROM that concern work-related items/total number of items of the PROM

A major shortcoming of the previously mentioned PROMs is that they only indicate the presence of limitations that patients experience while performing their job, but do not identify what these specific limitations are. Furthermore, it is not possible to identify to what degree these patients experience limitations since these PROMs are specifically developed for hand-wrist, elbow or shoulder disorders only, while patients with these disorders often suffer from limitations in the other parts of the upper extremity (Picavet and Schouten 2003). Therefore, clinicians and patients seem in need of a PROM that assesses the amount of limitations that are specifically work related, and that enhances communication between healthcare providers within and outside the hospital. Finally, such a PROM might be used to evaluate the effects of treatment on limitations in both individual workers and groups. The aims of this study were to develop a PROM specific for work-related limitations due to upper extremity disorders and to assess its content validity.

## Methods

### Target population

The target population consisted of patients between 18 and 65 years old with upper extremity musculoskeletal disorders visiting an orthopaedic outpatient clinic. They had to be part of the working population and have a job at the time of presentation and during treatment of the disease. Patients had to be able to understand the Dutch language and be able to read the questionnaire.

### Development of the WORQ-UP

In the present study, we used the criteria as formulated by Terwee et al. (2007) regarding quality criteria for the development and evaluation of health status questionnaires. These criteria were based on existing guidelines and on consensus within their research group.

Our research team consisted of an orthopaedic surgeon with experience in upper limb surgery, an orthopaedic hand surgeon specialised in upper limb surgery, an orthopaedic surgeon in training (Ph.D. candidate), a professor in occupational diseases and a human movement scientist working as an assistant professor with experience in the field of origin and prevention of work-related disorders, focused on musculoskeletal disorders. The team identified a set of work-related activities that would likely be affected by symptoms of upper extremity disorders. The items were carefully selected using the specific knowledge of our research group regarding work-related diseases and the influence upper extremity disorders might have on the ability to work. The existing PROMs used in clinical orthopaedic care were studied extensively to identify possible missing items. An overview of these PROMs can be found in Table 1. A selection of the collected items was made in a process of five consensus meetings.

### Determining content validity

According to the COSMIN (COnsensus-based Standards for the selection of health Measurement INstruments) taxonomy, content validity is the degree to which the content of a health-related patient-reported outcomes (HR-PRO) instrument is an adequate reflection of the construct to be measured (Guyatt et al. 1993; Terwee et al. 2007). To define and assess the content validity of the WORQ-UP, three steps were performed.

#### Step 1: patients’ remarks regarding the first version of the WORQ-UP

**Fig. 1:**
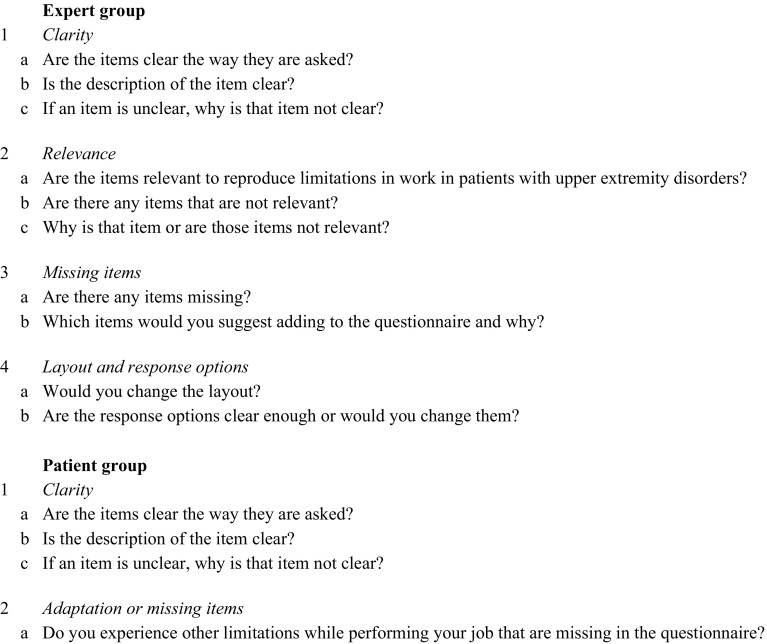
Interview questions for expert and patient interviews

A group of at least three randomly selected patients per body part (hand-wrist, elbow or shoulder) from our outpatient clinic was interviewed. Patients were selected as consecutive samples from the orthopaedic upper limb outpatient clinic. Patients had to meet the same inclusion criteria as the previously formulated target population. The aim of the interviews was to determine whether the questionnaire was clear, easy to understand, and if patients had any suggestions for items to be added or removed. Furthermore, the filling in and answering of the questionnaire was discussed. The interview questions are described in Fig. 1.

Interviews were held by researcher BA. The interviews were performed individually and took between 15 and 25 min per patient. The comments from patients were collected for each of the items. Comments were sorted out following the structure in Fig. 1. If comments from different patients were the same, we put them together to one single comment. The data were presented to the research group using MS PowerPoint.

#### Step 2: expert interviews

Professionals in work-related and clinical care for patients with upper extremity musculoskeletal disorders were also interviewed, using semi-structured (telephone) interviews. The group of experts consisted of physiotherapists, insurance physicians, occupational health physicians, rehabilitation physicians and orthopaedic surgeons. A minimum of three experts from each healthcare field were included. The experts could join if they met one or more of the following criteria: specialty in the field of upper extremity musculoskeletal disorders or, in work-related complaints of the upper extremity. Experts should treat patients with upper limb disorders at least one day in a week or be active in the field of research of work-related upper extremity disorders. Experts were randomly selected from different clinics throughout the Netherlands and Belgium. They were approached via email with an invitation to participate. If the expert agreed to participate, an appointment was made for the interview. The experts were contacted by telephone. The interviews were audio-recorded with permission from the experts. This was done to increase the reproducibility and comparability of answers of each participant and to give both participants and researcher the opportunity to elaborate on the topics that were discussed. Recorded data from the interviews were also used to evaluate the exact answers in a later phase of the study and to carefully register the interview data.

To collect the opinions in a structured and efficient way, the research group developed an interview containing the following items about the questionnaire: clarity, relevance, possible missing items, response options and layout. Furthermore, the interpretability of the items was discussed with the experts. The exact questions of the interview can be found in Fig. 1.

The interviews were held in the period from November 2013 until February 2014 by researcher BA. The results were collected in a database using MS Excel. After the interviews with the experts, researcher BA presented the detailed experts’ opinions and the main conclusions in three meetings with the research group. BA, described and grouped the qualitative data, the other members of the research group gave feedback. Comments were collected per healthcare field. If comments were similar, they were deleted to simplify the final table as much as possible. Per item, the comments were lined out for each healthcare field. Per item, we discussed all comments from all the experts.

Possible outcomes of the discussion were that an item was added, amended or deleted. Comments on the layout and interpretability of the items were discussed. This was done until consensus of the modifications to the WORQ-UP was reached among the research group.

#### Step 3: patients’ remarks regarding the second version of the WORQ-UP

Another group of at least three randomly selected patients per body part (hand-wrist, elbow or shoulder) from our outpatient clinic was interviewed. Patients had to meet the same inclusion criteria as mentioned before. The aim of these interviews was to discuss the layout and the content of the second version of the WORQ-UP. The same interview questions were used as shown in Fig. 1. Possible important and useful remarks were discussed in the research group and the WORQ-UP was adapted into the final version.

After definitive item selection, the questionnaire was translated from Dutch into English and back from English to Dutch by an external translating bureau allied to a university in the Netherlands. The translation was done to ensure consensus in the translation to an English language journal and to avoid bias from the research group. Both versions can be found in Tables 3 and 4, respectively.

## Results

### Development of the WORQ-UP

The research group composed the first version of the WORQ-UP that can be found in the first column of Table 2. The questionnaire consisted of 18 items. In the first version, patients were asked to fill in how difficult it was to perform several activities while performing their job (caused by symptoms of the affected upper extremity). The question was formulated in the following manner:

**Table 2:** Results from patient interviews before expert interviews

No	Item	Patient response before experts interviews (step 1) (*n* = 14)	
		Clarity	Adaptation/missing items
1	Lifting and carrying objects weighing more than 5 kg at between knee height and chest height	Clear	None
2	Lifting objects weighing more than 5 kg at or above shoulder height	Yes, but difficult to estimate how much 5 kg is	Add an example to clarify
3	Pushing and pulling objects weighing more than 25 kg	Clear	None
4	Working with your hands underneath knee height	Clear	None
5	Reaching with arms and hands	Clear	None
6	Working above shoulder height	Unclear how high and how much weight is meant	Add an example to clarify
7	Performing rapid and repetitive arm movements	Unclear what is meant	Adapt the question or add an example
8	Picking up small objects	Clear	None
9	Writing or making notes with a pen	Clear	None
10	Using your hand to exert force	Unclear what is meant Too broadly interpretable	Specify the question
11	Using hand tools (e.g. a hammer, brush or pliers)	Clear	Add question about household tasks
12	Doing manual work with machines (e.g. drills, torch or grinder)	Not applicable to many patients	
13	Driving a vehicle (e.g. a truck, van or car)	To and from work or driving a vehicle as part of the job?	Add an extra item with driving to and from work
14	Using a keyboard and/or mouse	Clear	None
15	Operating a smartphone or tablet with a touch screen	Clear	None
16	Climbing stairs	Not relevant according to majority of patients	Delete this item
17	Climbing or clambering up a ladder or scaffolding	Clear	None
18	Rising from a chair	Not relevant according to majority of patients	Delete this item

During the past week, how difficult was it for you or how difficult would it have been for you to perform the following activities during your work? This concerns the affected (hand/wrist OR elbow OR shoulder). If you rarely or never perform the described activity during your work, place a cross against the 0 = not applicable category.

Patients had to rate their difficulty on a six point scale: 0 = not applicable (in my job), 1 = not at all, 2 = slightly, 3 = moderately, 4 = very, 5 = extremely, or I can’t do this.

After examining the existing PROMs (Table 1), the research team proposed the following four criteria: exertion, dexterity, handling tools & equipment, and mobility. As a logical consequence, the items contained: activities that represent exertion (e.g. ‘lifting objects weighing more than 5 kg above shoulder height’, ‘lifting and carrying objects weighing more than 5 kg between knee height and chest height’ and ‘pushing and pulling objects weighing more than 25 kg’); activities that represent dexterity such as ‘picking up small objects’, ‘using hand tools’, ‘using a keyboard and/or mouse’ and ‘operating a smartphone or tablet with a touch screen’. Also items that represent handling tools and equipment such as ‘doing manual work with machines (e.g. drills, torch or grinder)’ and ‘using hand tools’ were included. Mobility was also considered as activities that can be compromised due to upper extremity disorders. Therefore, the item ‘driving a vehicle (e.g. a truck, van or car)’ was included. For a more general impression of movement, the items ‘climbing stairs’ and ‘rising from a chair’ were included.

According to the research group, this version included the items most likely to be affected in patients with upper extremity disorders.

### Determining content validity

#### Step 1: patients’ remarks regarding the first version of the WORQ-UP

The group of patients consisted of five patients with wrist/hand problems, five patients with complaints of the elbow and four patients with complaints of the shoulder (mean age 50 (31–60), 50% men). Patients indicated that almost half of the items (44% = 8/18) were difficult to understand. The most important remarks and suggestions included specific limitations for their particular jobs. Some of the items were too broad to understand. For example the item: ‘Exert force with the hand’. Depending on the background of the patient, there was a difference in interpretation. Some patients thought ‘exert force with the hand’ would be for example hitting an object, whereas others thought more about squeezing or wringing. The items ‘climbing stairs’ and ‘rising from a chair’ were considered not distinguishing enough for upper extremity disorders. Three patients indicated that their back problems would influence those activities. Also a significant portion of the patients indicated that it was not clear whether ‘driving a vehicle’ should be interpreted as work-related transport or in terms of commuting. The other remarks can be found in Table 2.

#### Step 2: expert interviews

A total of 48 experts were approached to participate in our study. Four of the professionals did not consider themselves to be an expert according to our selection criteria. Twenty-one experts agreed to participate. Twenty-three experts did not respond to the invitation. The average time for an interview was 21 min. All experts were able to answer the questions. The group of experts that was interviewed consisted of five physiotherapists specialised in work-related upper extremity disorders, five insurance physicians, four occupational health physicians, three rehabilitation physicians and four orthopaedic surgeons [10 men, mean age 45.8 (SD = 8.5) and mean years of experience 15.5 (SD = 9.9)]. Remarks on the items were categorised into three main groups (clarity, relevancy and missing items) in line with the interview questions. An overview of expert opinions can be found in Appendix 1. After the expert interviews, items were adapted according to the following possibilities: items were added with an example without adapting the item (*n* = 5); items were adapted without adding an example (*n* = 4); and items were adapted and an example was added (*n* = 2). Two completely new items were added, three items were not changed and three items were deleted.

##### Items were added with an example without adapting the item

Most of the examples that have been added to the five items (item numbers 1, 7, 8, 10 and 12) were necessary because of multiple possibilities of interpretation of the item. The example ‘e.g. a shopping bag containing five one-litre cartons of milk or soft drinks’ was added to the item ‘lifting and carrying objects weighing more than 5 kg at between knee height and chest height’ to clarify it for patients. Also ‘picking up small objects’ was not specific enough because it was unclear whether the small objects were heavy or not. Therefore, the example ‘e.g. a pen from a table’ was added.

##### Items were adapted without adding an example

The items 5, 9, 14 and 17 were adapted. For example ‘reaching with arms and hands’ was changed into ‘reaching forward with outstretched arms’ because of the fact that reaching is broadly interpretable and influenced. For shoulder diseases, for example, it usually makes a difference whether a patient reaches forward, below or above the shoulder for the severity of the complaints.

##### Items were adapted and an example was added

Items 2, 3 and 11 were adapted and also an example was added. Item 2 was adapted by reducing the amount of weight (2 kg) and also the example ‘putting a heavy binder into a cupboard’ was added. The item ‘pushing and pulling objects weighing more than 25 kg’ raised questions among the experts because it was unclear whether the objects were for example on wheels or not. So, it was adapted to ‘pushing and pulling wheeled devices weighing more than 60 kg (e.g. a full container/wheelie bin)’.

##### New items

Two completely new items were added by the research group to the original questionnaire after consideration of experts’ opinion. The first item is ‘travelling to and from your work (e.g. by car, scooter or moped)’ because a reason for absenteeism might be that employees are not able to commute. Another item missing according to the majority (*n* = 15; 71%) of experts was an item about the inability to work with machines that cause vibrations. The item ‘using heavy equipment that causes vibration (e.g. a hammer drill or demolition hammer’) was added.

##### Items that were not changed

Items 6, 13 and 15 were not changed from the original WORQ-UP.

##### Deleted items

The item ‘climbing stairs’ was deleted from the questionnaire because the majority of the experts (*n* = 19; 91%;) agreed that the back and lower extremity were too much involved when climbing stairs. Therefore, the item was considered to be not specific enough for upper extremity disorders.

Most experts (*n* = 19; 91%) agreed that the majority of patients with complaints of the upper extremity would not use the upper extremity when rising from a chair. Therefore, this item was also considered to be not specific enough for upper extremity disorders and deleted from the questionnaire. Fourteen experts (67%) agreed that working with hands beneath knee height would also bias the results because the lower extremity and back are involved in that activity. Therefore, the item ‘working with your hands underneath knee height’ was deleted.

#### Step 3: patients’ opinion of the second version of the WORQ-UP

The group of patients consisted of five patients with wrist/hand problems, three patients with complaints of the elbow and four patients with complaints of the shoulder (mean age 45 (21–61), 50% men). No remarks resulted in amendments to the 17 items. In general, this version of the questionnaire was found to be understandable and most of the patients’ suggestions for changes or additions were based on specifications of their own jobs and not appropriate for the questionnaire.

After the modifications mentioned before, the final version of the WORQ-UP consists of 17 items (Tables 3, 4). Translation of the WORQ-UP from Dutch into English and English into Dutch did not reveal any problems. The only word translators had discussion about was the Dutch word ‘steiger’. The translators first translated this using the word ‘plateau’. We felt that the word ‘plateau’ was not correct in this context and changed it into ‘scaffolding’.

**Table 3:** WORQ-UP in Dutch

	Nvt	Geen	Gering	Matig	Veel	Erg veel, of Kan ik niet
Tillen en dragen van voorwerpen zwaarder dan 5 kg tussen knie- en borsthoogte, bijvoorbeeld een boodschappentas met 5 literpakken melk of frisdrank	0	1	2	3	4	5
Tillen van voorwerpen zwaarder dan 2 kg boven schouderhoogte, bijvoorbeeld een zware ordner in de kast zetten	0	1	2	3	4	5
Duwen en trekken van rollend materieel zwaarder dan 60 kg, bijvoorbeeld een volle container/kliko	0	1	2	3	4	5
Reiken met gestrekte armen naar voren	0	1	2	3	4	5
Werken boven schouderhoogte	0	1	2	3	4	5
Snel herhaalde armbewegingen uitvoeren, bijvoorbeeld tijdens post sorteren of lopende band werk doen	0	1	2	3	4	5
Kleine voorwerpen pakken, bijvoorbeeld een pen van de tafel	0	1	2	3	4	5
Schrijven met een pen/potlood	0	1	2	3	4	5
Kracht uitoefenen met de hand, zoals knijpen/wringen/kneden	0	1	2	3	4	5
Werken met handgereedschappen zoals een schroevendraaier	0	1	2	3	4	5
Handmatig werken met machines of apparaten zoals boormachine of slijptol	0	1	2	3	4	5
Besturen van een voertuig zoals vrachtwagen, bestelbus of auto	0	1	2	3	4	5
Werken met een toetsenbord en/of muis	0	1	2	3	4	5
Smartphone of tablet bedienen met een aanraakbeeldscherm (touch screen)	0	1	2	3	4	5
Klimmen op een ladder of stellage	0	1	2	3	4	5
Werken met zware apparaten die trillingen veroorzaken zoals een klopboormachine of breekhamer	0	1	2	3	4	5
Het reizen van en naar uw werk, bijvoorbeeld met de auto, scooter of bromfiets	0	1	2	3	4	5

*Hoeveel moeite heeft u* of zou u in de afgelopen week hebben gehad met het uitvoeren van de volgende activiteiten in uw werk? Het gaat *over de hand/pols OF elleboog OF schouder waar u de klachten* heeft. Wanneer u de genoemde activiteit zelden tot nooit in uw werk uitvoert, dan kruist u de categorie ‘Nvt = Niet van toepassing’ aan

**Table 4:** WORQ-UP in English

	NA	Not at all	Slightly	Moderately	Very	Extremely, or I can’t do this
Lifting and carrying objects weighing more than 5 kg at between knee height and chest height (e.g. a shopping bag containing five one-litre cartons of milk or soft drinks)	0	1	2	3	4	5
Lifting objects weighing more than 2 kg above shoulder height (e.g. putting a heavy binder into a cupboard)	0	1	2	3	4	5
Pushing and pulling wheeled devices weighing more than 60 kg (e.g. a full container/wheelie bin)	0	1	2	3	4	5
Reaching forward with outstretched arms	0	1	2	3	4	5
Working above shoulder height	0	1	2	3	4	5
Performing rapid and repetitive arm movements (e.g. sorting the post or doing assembly line work)	0	1	2	3	4	5
Picking up small objects (e.g. a pen from a table)	0	1	2	3	4	5
Writing with a pen/pencil	0	1	2	3	4	5
Using your hand to exert force (e.g. squeezing/wringing/kneading)	0	1	2	3	4	5
Using hand tools (e.g. a screwdriver)	0	1	2	3	4	5
Doing manual work with machines (e.g. a drill or a grinder)	0	1	2	3	4	5
Driving a vehicle (e.g. a truck, van or car)	0	1	2	3	4	5
Using a keyboard and/or mouse	0	1	2	3	4	5
Operating a smartphone or tablet by means of a touch screen	0	1	2	3	4	5
Climbing up a ladder or scaffolding	0	1	2	3	4	5
Using heavy equipment that causes vibration (e.g. a hammer drill or demolition hammer)	0	1	2	3	4	5
Travelling to and from your work (e.g. by car, scooter or moped)	0	1	2	3	4	5

During the past week, *how difficult was it for you* or how difficult would it have been for you to perform the following activities during your work? This concerns *the affected hand/wrist OR elbow OR shoulder*. If you rarely or never perform the described activity during your work, place a cross against the “NA = not applicable” category

## Discussion

Our research group developed a 17-item PROM (WORQ-UP) to assess the degree of specific work-related limitations that actively working patients with upper extremity disorders might experience. The final version of the WORQ-UP consists of 17 different items regarding limitations in work. The items include four main groups of activities: exertion, dexterity, handling tools & equipment, and mobility.

A strength of this study is that we used feedback from both patients and experts to develop the WORQ-UP. The importance of their involvement is apparent from the fact that our first version was greatly changed after comments from patients and experts in the field. This is in line with the quality criteria for content validity as proposed by Terwee et al. (2007). Furthermore, the input of patients and experts from the target population gave us the opportunity to develop an understandable and meaningful questionnaire that might be valued by both patients and healthcare workers. In addition, we expect the WORQ-UP to provide information on limitations that patients might experience before and after surgical and non-surgical treatment for upper extremity disorders. Therefore, responsiveness is one of the characteristics of the WORQ-UP we will study in the future.

As mentioned before, we consider a questionnaire in which patients are able to register the limitations they experience, to be the most useful instrument for monitoring work-related upper extremity complaints. An alternative might be to use performance-based tests. However, they are more difficult to incorporate in daily clinical practice while their usefulness might vary a lot between different types of work. Strength, for example, might be necessary for a blue-collar worker, but of less importance for a white-collar worker. In an ideal situation, the clinician should be able to combine several tests. One might combine the WORQ-UP with a performance-based test. In patients with physically demanding work, there are indications that combining performance-based tests, such as a lifting test, with self-reported work ability nearly doubles the explained variance for sustainable return to work (Kuijer et al. 2012).

In the development of a new questionnaire, 50–100 patients should be interviewed to determine all areas of dysfunction according to Guyatt et al. (1986) followed by another round of interviews with 50–100 different patients to assess the importance of the selected items. An alternative approach proposed by the same authors is to interview content-area experts (e.g. physicians, physiotherapists) and to use a small number of patients who are affected by the specific disease. For the development of the WORQ-UP, we chose to perform this second approach for item selection for the WORQ-UP since the questionnaire should be both of value for patients to rate their limitations and experts to decide whether additional work-directed care is needed to address the work limitations. In addition, we decided not to start out with a large number of items but to carefully select the items since a large pool of items will not automatically guarantee a better content validity (Terwee et al. 2007). Another reason to choose this approach is the fact that the area of interest includes work-related limitations only. We had no interest in, for example physical status, limitations in general daily activities and sport-related limitations. The variation in items was restricted equally. Furthermore, experts were given the opportunity to name missing items and provide missing areas where limitations might be seen in patients with upper extremity disorders.

In the second phase of development, the assessment of the content validity, we further developed the questionnaire together with 21 experts and 26 patients from the target population. The WORQ-UP is widely supported by different professionals. When compared to currently available questionnaires, the WORQ-UP is designed not only to identify the presence of limitations that patients with upper extremity musculoskeletal disorders experience while performing activities in their job. It also identifies the nature and severity of these limitations. Existing PROMs are compared with the WORQ-UP regarding limitations in work-related activities in Table 1  (Chung et al. 1998; Dawson et al. 1996; Hollinshead et al. 2000; Hudak et al. 1996; Kirkley et al. 1998, 2003; Lo et al. 2001; MacDermid et al. 1998; Michener et al. 2002). Since the WORQ-UP differs from other PROMs, it may be considered as an expansion in patient-related outcome measures, rather than a competing PROM. An existing PROM to assess work-related problems of the upper extremity is the DASH-score. It can be used for upper extremity disorders in general and has a separate work module consisting four questions (Dale et al. 2015). The WORQ-UP, however, is more specific to the working situation. It contains 17 items that measure the nature and severity of work-related limitations in patients with upper extremity disorders. Furthermore, it shows what those limitations are and to what degree they are present in their work, according to patients. The WORQ-UP is able to determine severity due to the five-point scale (0 = not applicable). Using the WORQ-UP at various moments, patients are able to indicate how severe their complaints are in performing their jobs, while clinicians will be able to monitor changes during treatment.

Limitations of this study are that the interviews were not conducted in groups. Focus group discussions, especially in the clinician and second round of patient interviews, might have led to more extensive and/or different input from the patient and expert groups. Another limitation is that we did not select the patient groups from different professions. We included patients that had a job at the moment of inclusion; but did not select an equal amount of blue-collar workers and white-collar workers. Furthermore for this study, patients with cervical spine disease were not included. One could debate that the WORQ-UP should be tested in these patients too since cervical spine disease might give rise to upper limb complaints. This would be an interesting topic for further research.

As a next step, we intend to investigate whether the WORQ-UP is reliable, reproducible, has a good construct validity and is responsive. After that we would like to calculate sum scores to enhance the clinical relevance.

This paper describes the development of a new questionnaire for work-related upper extremity complaints. It has been constructed after consulting experts from different medical and research fields. All experts are actively involved in work-related patient care. Our hopes are that the WORQ-UP will enhance communication between such experts in the near future, thereby improving care for patients that experience limitations in work and participation. The WORQ-UP provides medical and paramedical specialists with a means to communicate about the limitations in work, in patients with upper extremity disorders, in a more uniform way. Furthermore, these specialists will have the opportunity to register the amount and type of limitations in work that the patient experiences allowing development of a more personal treatment regarding the limitations in work.

We expect the WORQ-UP to be a useful tool in assessing work-related limitations in upper extremity musculoskeletal disorders and to streamline the interaction between the curative and the occupational health sectors. The next step will include the further investigation of internal consistency, reliability, validity and responsiveness.

## Conclusions

A new 17-item PROM to assess limitations in work in patients with upper extremity musculoskeletal disorders named WORQ-UP was developed. The content validity of the WORQ-UP was assessed in close cooperation with patients from the target population and a variety of health experts from the field, which therefore increases the likelihood that the WORQ-UP will be valued by both patients and healthcare workers.

## Electronic supplementary material

Below is the link to the electronic supplementary material.

Supplementary material 1 (DOCX 24 kb)
