# Supplementary material for: Development of a novel WOrk-Related Questionnaire for UPper extremity disorders (WORQ-UP)
Source: Int Arch Occup Environ Health. 2017 Jul 11;90(8):823–33. doi: 10.1007/s00420-017-1246-7 (PMC5640741; doi:10.1007/s00420-017-1246-7)
Supplement: Supplementary file 1 — Supplementary material 1 (DOCX 24 kb) [file 420_2017_1246_MOESM1_ESM.docx]

**Appendix 1 Results from expert interviews (part1)**

| **No** | **Item** | **Physiotherapists**  **(n=5)** | | | |  |  | **Insurance physicians (n=5)** | | | |  |  | **Occupational health physicians (n=4)** | | | | | |
| --- | --- | --- | --- | --- | --- | --- | --- | --- | --- | --- | --- | --- | --- | --- | --- | --- | --- | --- | --- |
|  |  |  |  |  |  |  |  |  |  |  |  |  |  |  |  |  |  |  |  |
|  |  | clear |  | relevant | | missing | | clear |  | relevant | | missing | | clear |  | relevant | | missing | |
|  |  | yes | no | yes | no | yes | no | yes | no | yes | no | yes | no | yes | no | yes | no | yes | no |
| 1 | Lifting and carrying objects weighing more than | 4 | 1 | 3 | 2 | 1 | 4 | 4 | 1 | 3 | 2 | 2 | 3 | 3 | 1 | 2 | 2 | 2 | 2 |
|  | 5 kg at between knee height and chest height |  |  |  |  |  |  |  |  |  |  |  |  |  |  |  |  |  |  |
| 2 | Lifting objects weighing more than 5 kg | 4 | 1 | 1 | 4 | 1 | 4 | 4 | 1 | 3 | 2 | 2 | 3 | 3 | 1 | 4 | 0 | 1 | 3 |
|  | at or above shoulder height |  |  |  |  |  |  |  |  |  |  |  |  |  |  |  |  |  |  |
| 3 | Pushing and pulling objects weighing | 4 | 1 | 4 | 1 | 4 | 1 | 4 | 1 | 3 | 2 | 2 | 3 | 3 | 1 | 4 | 0 | 1 | 3 |
|  | more than 25 kg |  |  |  |  |  |  |  |  |  |  |  |  |  |  |  |  |  |  |
| 4 | Working with your hands | 4 | 1 | 4 | 1 | 2 | 3 | 3 | 2 | 4 | 1 | 1 | 4 | 4 | 0 | 4 | 0 | 0 | 4 |
|  | underneath knee height |  |  |  |  |  |  |  |  |  |  |  |  |  |  |  |  |  |  |
| 5 | Reaching with arms and hands | 1 | 4 | 4 | 1 | 2 | 3 | 2 | 3 | 5 | 0 | 2 | 3 | 1 | 3 | 1 | 2 | 3 | 1 |
|  |  |  |  |  |  |  |  |  |  |  |  |  |  |  |  |  |  |  |  |
| 6 | Working above shoulder height | 5 | 0 | 0 | 0 | 0 | 5 | 4 | 1 | 5 | 0 | 1 | 4 | 4 | 0 | 4 | 0 | 0 | 4 |
|  |  |  |  |  |  |  |  |  |  |  |  |  |  |  |  |  |  |  |  |
| 7 | Performing rapid and | 2 | 3 | 4 | 1 | 3 | 2 | 4 | 1 | 4 | 1 | 2 | 3 | 3 | 1 | 3 | 1 | 1 | 3 |
|  | repetitive arm movements |  |  |  |  |  |  |  |  |  |  |  |  |  |  |  |  |  |  |
| 8 | Picking up small objects | 4 | 1 | 4 | 1 | 1 | 4 | 4 | 1 | 4 | 1 | 1 | 4 | 4 | 0 | 4 | 0 | 1 | 3 |
|  |  |  |  |  |  |  |  |  |  |  |  |  |  |  |  |  |  |  |  |
| 9 | Writing or making notes with a pen | 5 | 0 | 5 | 0 | 0 | 5 | 5 | 0 | 5 | 0 | 0 | 5 | 3 | 1 | 4 | 0 | 1 | 3 |
|  |  |  |  |  |  |  |  |  |  |  |  |  |  |  |  |  |  |  |  |
| 10 | Using your hand to exert force | 4 | 1 | 4 | 1 | 0 | 5 | 3 | 2 | 5 | 0 | 2 | 3 | 1 | 3 | 4 | 0 | 2 | 2 |
|  |  |  |  |  |  |  |  |  |  |  |  |  |  |  |  |  |  |  |  |
| 11 | Using hand tools (e.g. a hammer, brush or | 3 | 2 | 5 | 0 | 2 | 3 | 4 | 1 | 5 | 0 | 3 | 2 | 2 | 2 | 3 | 1 | 3 | 1 |
|  | pliers) |  |  |  |  |  |  |  |  |  |  |  |  |  |  |  |  |  |  |
| 12 | Doing manual work with machines (e.g. drills, | 4 | 0 | 5 | 0 | 1 | 4 | 5 | 0 | 5 | 0 | 1 | 4 | 3 | 1 | 4 | 0 | 1 | 3 |
|  | torch or grinder) |  |  |  |  |  |  |  |  |  |  |  |  |  |  |  |  |  |  |
| 13 | Driving a vehicle (e.g. a truck, van or car) | 5 | 0 | 5 | 0 | 2 | 3 | 4 | 1 | 5 | 0 | 0 | 5 | 4 | 0 | 4 | 0 | 0 | 4 |
|  |  |  |  |  |  |  |  |  |  |  |  |  |  |  |  |  |  |  |  |
| 14 | Using a keyboard and/or mouse | 5 | 0 | 5 | 0 | 0 | 5 | 5 | 0 | 5 | 0 | 1 | 4 | 4 | 0 | 4 | 0 | 0 | 4 |
|  |  |  |  |  |  |  |  |  |  |  |  |  |  |  |  |  |  |  |  |
| 15 | Operating a smartphone or tablet | 5 | 0 | 5 | 0 | 1 | 4 | 5 | 0 | 5 | 0 | 0 | 5 | 4 | 0 | 4 | 0 | 0 | 4 |
|  | with a touch screen |  |  |  |  |  |  |  |  |  |  |  |  |  |  |  |  |  |  |
| 16 | Climbing stairs | 5 | 0 | 0 | 5 | 0 | 5 | 5 | 0 | 0 | 5 | 0 | 5 | 1 | 3 | 0 | 4 | 1 | 3 |
|  |  |  |  |  |  |  |  |  |  |  |  |  |  |  |  |  |  |  |  |
| 17 | Climbing or clambering up a | 4 | 1 | 5 | 0 | 0 | 5 | 4 | 1 | 5 | 0 | 0 | 5 | 4 | 0 | 4 | 0 | 0 | 4 |
|  | ladder or scaffolding |  |  |  |  |  |  |  |  |  |  |  |  |  |  |  |  |  |  |
| 18 | Rising from a chair | 5 | 0 | 0 | 5 | 0 | 5 | 5 | 0 | 0 | 5 | 0 | 5 | 4 | 0 | 0 | 4 | 0 | 4 |
|  |  |  |  |  |  |  |  |  |  |  |  |  |  |  |  |  |  |  |  |

The digit represents the number of experts that agreed whether the item was clear or not, relevant or not and if an item was missing.

**Appendix 1 Results from expert interviews (part2)**

| **No** | **Item** | **Rehabilitation physicians** | | | | |  | **Orthopaedic surgeons** | | | |  |  |
| --- | --- | --- | --- | --- | --- | --- | --- | --- | --- | --- | --- | --- | --- |
|  |  | **(n=3)** |  |  |  |  |  | **(n=4)** |  |  |  |  |  |
|  |  | clear |  | relevant | | missing | | clear |  | relevant | | Missing | |
|  |  | yes | no | yes | No | yes | no | yes | no | yes | no | yes | no |
| 1 | Lifting and carrying objects weighing more than | 2 | 1 | 2 | 1 | 2 | 1 | 3 | 1 | 4 | 0 | 1 | 3 |
|  | 5 kg at between knee height and chest height |  |  |  |  |  |  |  |  |  |  |  |  |
| 2 | Lifting objects weighing more than 5 kg | 1 | 2 | 1 | 2 | 1 | 2 | 2 | 2 | 4 | 0 | 2 | 2 |
|  | at or above shoulder height |  |  |  |  |  |  |  |  |  |  |  |  |
| 3 | Pushing and pulling objects weighing | 3 | 0 | 3 | 0 | 0 | 3 | 3 | 1 | 3 | 1 | 1 | 3 |
|  | more than 25 kg |  |  |  |  |  |  |  |  |  |  |  |  |
| 4 | Working with your hands | 2 | 1 | 1 | 2 | 1 | 2 | 2 | 2 | 1 | 3 | 1 | 3 |
|  | underneath knee height |  |  |  |  |  |  |  |  |  |  |  |  |
| 5 | Reaching with arms and hands | 1 | 2 | 2 | 1 | 2 | 1 | 4 | 0 | 4 | 0 | 0 | 4 |
|  |  |  |  |  |  |  |  |  |  |  |  |  |  |
| 6 | Working above shoulder height | 3 | 0 | 3 | 0 | 0 | 3 | 4 | 0 | 4 | 0 | 0 | 4 |
|  |  |  |  |  |  |  |  |  |  |  |  |  |  |
| 7 | Performing rapid and | 3 | 0 | 2 | 1 | 1 | 2 | 3 | 1 | 4 | 0 | 1 | 3 |
|  | repetitive arm movements |  |  |  |  |  |  |  |  |  |  |  |  |
| 8 | Picking up small objects | 2 | 1 | 3 | 0 | 2 | 1 | 2 | 2 | 4 | 0 | 2 | 2 |
|  |  |  |  |  |  |  |  |  |  |  |  |  |  |
| 9 | Writing or making notes with a pen | 3 | 0 | 3 | 0 | 0 | 3 | 4 | 0 | 4 | 0 | 1 | 3 |
|  |  |  |  |  |  |  |  |  |  |  |  |  |  |
| 10 | Using your hand to exert force | 2 | 1 | 3 | 0 | 1 | 2 | 2 | 2 | 3 | 1 | 2 | 2 |
|  |  |  |  |  |  |  |  |  |  |  |  |  |  |
| 11 | Using hand tools (e.g. a hammer, brush or | 1 | 1 | 1 | 1 | 1 | 1 | 3 | 1 | 4 | 0 | 1 | 3 |
|  | pliers) |  |  |  |  |  |  |  |  |  |  |  |  |
| 12 | Doing manual work with machines (e.g. drills, | 1 | 2 | 2 | 1 | 2 | 1 | 3 | 1 | 4 | 0 | 1 | 3 |
|  | torch or grinder) |  |  |  |  |  |  |  |  |  |  |  |  |
| 13 | Driving a vehicle (e.g. a truck, van or car) | 3 | 0 | 3 | 0 | 0 | 3 | 2 | 2 | 3 | 1 | 1 | 3 |
|  |  |  |  |  |  |  |  |  |  |  |  |  |  |
| 14 | Using a keyboard and/or mouse | 3 | 0 | 3 | 0 | 0 | 3 | 3 | 1 | 4 | 0 | 1 | 3 |
|  |  |  |  |  |  |  |  |  |  |  |  |  |  |
| 15 | Operating a smartphone or tablet | 3 | 0 | 3 | 0 | 0 | 3 | 1 | 1 | 4 | 0 | 2 | 2 |
|  | With a touch screen |  |  |  |  |  |  |  |  |  |  |  |  |
| 16 | Climbing stairs | 3 | 0 | 1 | 2 | 1 | 2 | 4 | 0 | 1 | 3 | 1 | 3 |
|  |  |  |  |  |  |  |  |  |  |  |  |  |  |
| 17 | Climbing or clambering up a | 2 | 1 | 2 | 1 | 1 | 2 | 4 | 0 | 4 | 0 | 0 | 4 |
|  | ladder or scaffolding |  |  |  |  |  |  |  |  |  |  |  |  |
| 18 | Rising from a chair | 3 | 0 | 1 | 2 | 1 | 2 | 2 | 2 | 1 | 3 | 0 | 4 |
|  |  |  |  |  |  |  |  |  |  |  |  |  |  |

The digit represents the number of experts that agreed whether the item was clear or not, relevant or not and if an item was missing.
